# Diversity of *Rickettsia* species in border regions of northwestern China

**DOI:** 10.1186/s13071-018-3233-6

**Published:** 2018-12-13

**Authors:** Shengnan Song, Chuangfu Chen, Meihua Yang, Shanshan Zhao, Baoju Wang, Sándor Hornok, Bolatkhan Makhatov, Kadyken Rizabek, Yuanzhi Wang

**Affiliations:** 1 Department of Veterinary Medicine, College of Animal & Science, Shihezi University, Shihezi, Xinjiang, Uygur Autonomous Region China; 2 Department of Forestry, College of Agriculture, Shihezi University, Shihezi, Xinjiang, Uygur Autonomous Region China; 3 Department of Basic Medicine, School of Medicine, Shihezi University, Shihezi, Xinjiang, Uygur Autonomous Region China; 4 Department of Infectious Diseases, Union Hospital of Tongji Medical College, Huazhong University of Science and Technology, Wuhan, China; 5 Department of Parasitology and Zoology, University of Veterinary Medicine, Budapest, Hungary; 6 Department of Anatomy, Physiology and Biochemistry, Kazakh National Agrarian University, Almaty, Kazakhstan; 7 Department of Food Engineering, Kazakh National Agrarian University, Almaty, Kazakhstan

**Keywords:** Fleas, Northwestern China, *Rickettsia*, Ticks

## Abstract

**Background:**

*Rickettsia* species belonging to the spotted fever group (SFG) cause infections in humans, domestic animals and wildlife. At least ten SFG *Rickettsia* species are known to occur in China. However, the distribution of rickettsiae in ticks and fleas in the border region of northwestern China have not been systematically studied to date.

**Results:**

A total of 982 ticks (*Rhipicephalus turanicus*, *Dermacentor marginatus*, *D. nuttalli* and *Haemaphysalis punctata*) and 5052 fleas (18 flea species from 14 species of wild mammals) were collected in ten and five counties, respectively, of Xinjiang Uygur Autonomous Region (northwestern China). Tick and flea species were identified according to morphological and molecular characteristics. Seven sets of primers for amplifying the 17-kDa antigen gene (17*-kDa*), citrate synthase gene (*glt*A), *16S* rRNA gene (*rrs*), outer membrane protein A and B genes (*omp*A, *omp*B), surface cell antigen 1 gene (*sca*1) and PS120-protein encoding gene (*gene* D) were used to identify the species of rickettsiae. Nine *Rickettsia* species have been detected, seven of them in ticks: *R*. *aeschlimannii*, *R*. *conorii*, *R*. *raoultii*, *Rickettsia sibirica*, *R*. *slovaca*, *R*. *massiliae* and “*Candidatus* R. barbariae”. In addition, *R*. *bellii* and two genotypes of a rickettsia endosymbiont (phylogenetically in an ancestral position to *R. bellii*) have been detected from flea pools.

**Conclusions:**

This study provides molecular evidence for the occurrence of several SFG rickettsiae in *Rhipicephalus turanicus*, *Dermacentor nuttalli* and *D. marginatus*. Furthermore, *R. bellii* and two ancestral rickettsia endosymbionts are present in fleas infesting wild rodents in the border regions of northwestern China. These data extend our knowledge on the diversity of rickettsiae in Central Asia.

**Electronic supplementary material:**

The online version of this article (10.1186/s13071-018-3233-6) contains supplementary material, which is available to authorized users.

## Background

Rickettsiae are obligate intracellular Gram-negative bacteria causing infection in humans, domestic animals and wildlife [1, 2]. Their vectors are typically ticks, fleas or mites, but rickettsiae were also shown to be present in several other arthropod groups [3, 4]. There is a great variety of clinical presentations of rickettsioses, and some pathogenic species, which cause debilitating diseases, are listed as bioterrorism agents [5]. Members of the genus *Rickettsia* are divided into four clades: spotted fever group (SFG); typhus group (TG); ancestral group (AG); and transitional group (TRG) [6]. To date, ten valid *Rickettsia* species have been detected in China [7–10]. In previous studies, seven species of rickettsiae (including *R*. *aeschlimannii*, *R*. *conorii*, *R*. *raoultii*, *Rickettsia sibirica*, *R*. *slovaca*, *R*. *massiliae* and “*Candidatus* R. barbariae”) were shown to be present in ticks or fleas in Xinjiang Uygur Autonomous Region (XUAR) [7, 8].

There is a great diversity of tick and flea species in XUAR, owing to the variability of geographical landscape and the availability of multiple vertebrate host species for these parasites [11, 12]. Therefore, the aim of this study was to systematically analyze the occurrence of *Rickettsia* species in ticks and fleas in the border region of XUAR.

## Methods

### Study area and sample collection

**Fig. 1:**
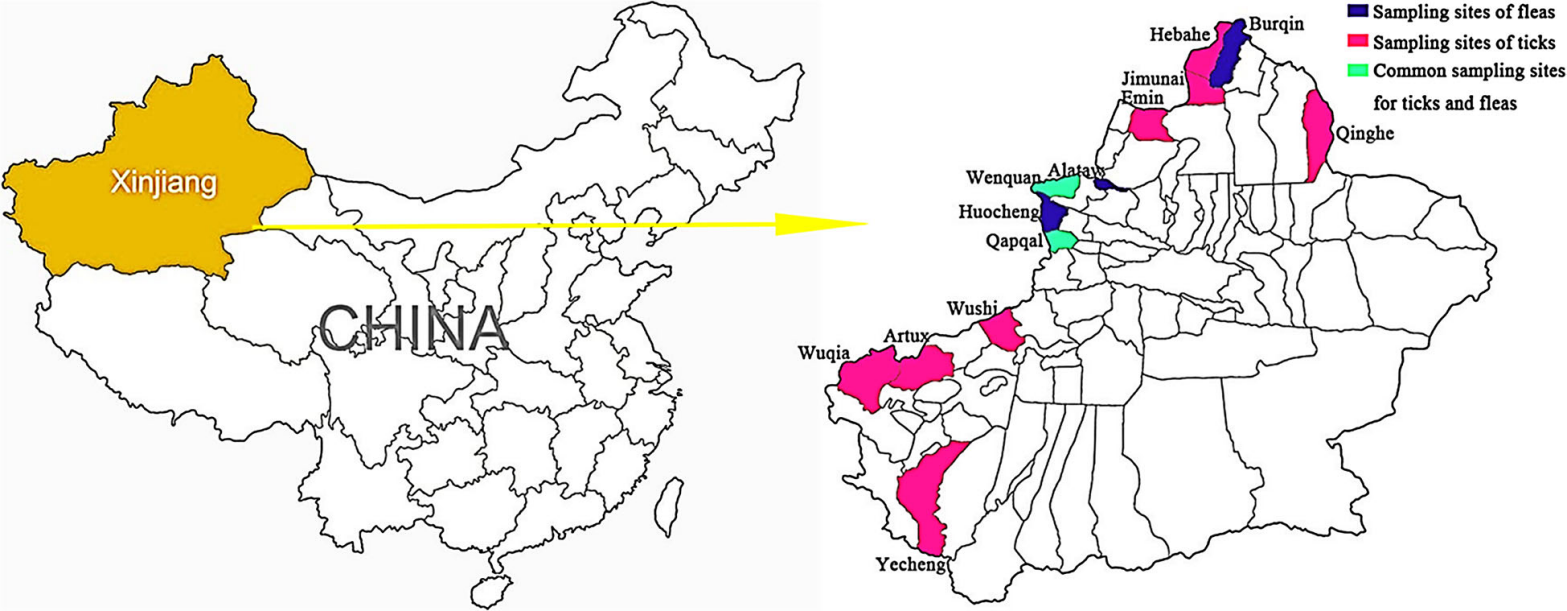
A map of the study area. Left: the People’s Republic of China. Right: the thirteen surveyed border counties in the XUAR

XUAR, located in northwestern China, covers 1.66 million square kilometers, and is bordered by eight countries (in the northeast Mongolia, northward the Russian Federation, in the northwest and west Kazakhstan, Kyrgyzstan, Tajikistan, and in the southwest Afghanistan, Pakistan and India) [13]. During the period between 2014 and 2016, late April to mid-May (coinciding with the peak activities of adult ticks), 982 adult ticks (675 males and 307 females) were collected from ten counties including Jimunai, Emin, Wenquan, Qapqal, Qinghe, Habahe, Wushi, Artux, Wuqia and Yecheng, while 5052 fleas were collected from Alataw, Burqin, Huocheng, Wenquan and Qapqal counties in the border area of XUAR (Fig. 1). The first six counties of tick sampling and the first three counties of flea sampling are located in the northern region, whereas the remaining counties are located in the southern part of XUAR. Jimunai, Emin, Wenquan, Qapqal, Artux and Huocheng counties are adjacent to Kazakhstan. Artux, Wushi and Wuqia counties are adjacent to Kyrgyzstan, Qinghe and Yecheng counties are neighboring on Mongolia and Pakistan, respectively, and Habahe county shares a border with Russia and Kazakhstan.

Ticks were collected from cattle and sheep after examining the entire body of each animal (including predilection sites such as ears, neck, armpits, thorax, abdomen, femurs, perianal region, etc.) [11, 14]. For flea sampling rodents were captured with Sherman traps (H.B. Sherman Traps, Tallahassee, Florida, USA), which were placed at the entrances of occupied burrows [15]. Each survey site included 150 traps that were checked twice a day. Each trap was removed before nightfall and replaced on the survey site the following day. The abundance and species of fleas were determined in each captured rodent. The animal fur was combed thoroughly until no additional fleas were recovered. After this, each rodent was released [16].

### Morphological and molecular identification of ticks

All ticks were identified morphologically according to previous reports [17, 18]. To confirm tick species, 80 specimens (5–10 for each tick species representing every sampling county), were used for molecular taxonomic analysis. The genomic DNA was extracted from each tick individually, using the TIANamp Genomic DNA Kit (TIANGEN, Beijing, China). All 80 tick DNA extracts analyzed based on partial mitochondrial [*12S rRNA*, *16S rRNA* and cytochrome *c* oxidase subunit 1 (*cox*1)] gene sequences [19, 20].

### Morphological and molecular identification of fleas

Fleas were identified morphologically using a compound microscope and observing key structures [21, 22]. Depending on the flea species, host and sampling site, every 1–15 fleas were pooled together for DNA extraction by using Isolate II Genomic DNA Kit (BioLine, Sydney, Australia) as previously described [23]. The DNA extracts of 70 flea pools were included in multi-locus sequence analysis using four genes, i.e. the *18S* ribosomal DNA (*18S* rDNA), *28S* ribosomal DNA (*28S* rDNA), *cox*2 and elongation 1-alpha (*EF-1a*) as described previously [24].

### Detection of rickettsial agents and sequence analysis

Seven sets of primers for amplifying the 17-kDa antigen gene (17*-kDa*), citrate synthase gene (*glt*A), *16S* rRNA gene (*rrs*), outer membrane protein A and B genes (*omp*A, *omp*B), surface cell antigen 1 gene (*sca*1) and PS120-protein encoding gene (*gene* D) were used to identify the species of rickettsia [25–27]. An additional genetic marker *17-kDa*^2^ was used to confirm the presence of the rickettsiae in fleas [28]. Sequence-confirmed rickettsia DNA amplified in our laboratory and double distilled water (Dongsheng, Guangzhou, China) were used as positive and negative controls, respectively. PCR products were purified using the TIANgel Midi Purification Kit (TIANGEN, Beijing, China) and cloned into the pGEM-T Easy vector and subjected to sequencing. A phylogenetic tree was constructed using the maximum-likelihood (ML) method with MEGA 6 software [29].

All sequences obtained in this study were compared with GenBank data using the nucleotide BLAST program (http://www.ncbi.nlm.nih.gov/BLAST/). Representative sequences including 22 from ticks, 78 from fleas, 84 from rickettsiae in ticks and 12 from rickettsiae in fleas have been deposited in the GenBank database (accession numbers shown in Additional file 1: Table S1, sections A, B, C, D, respectively).

## Results

**Fig. 2:**
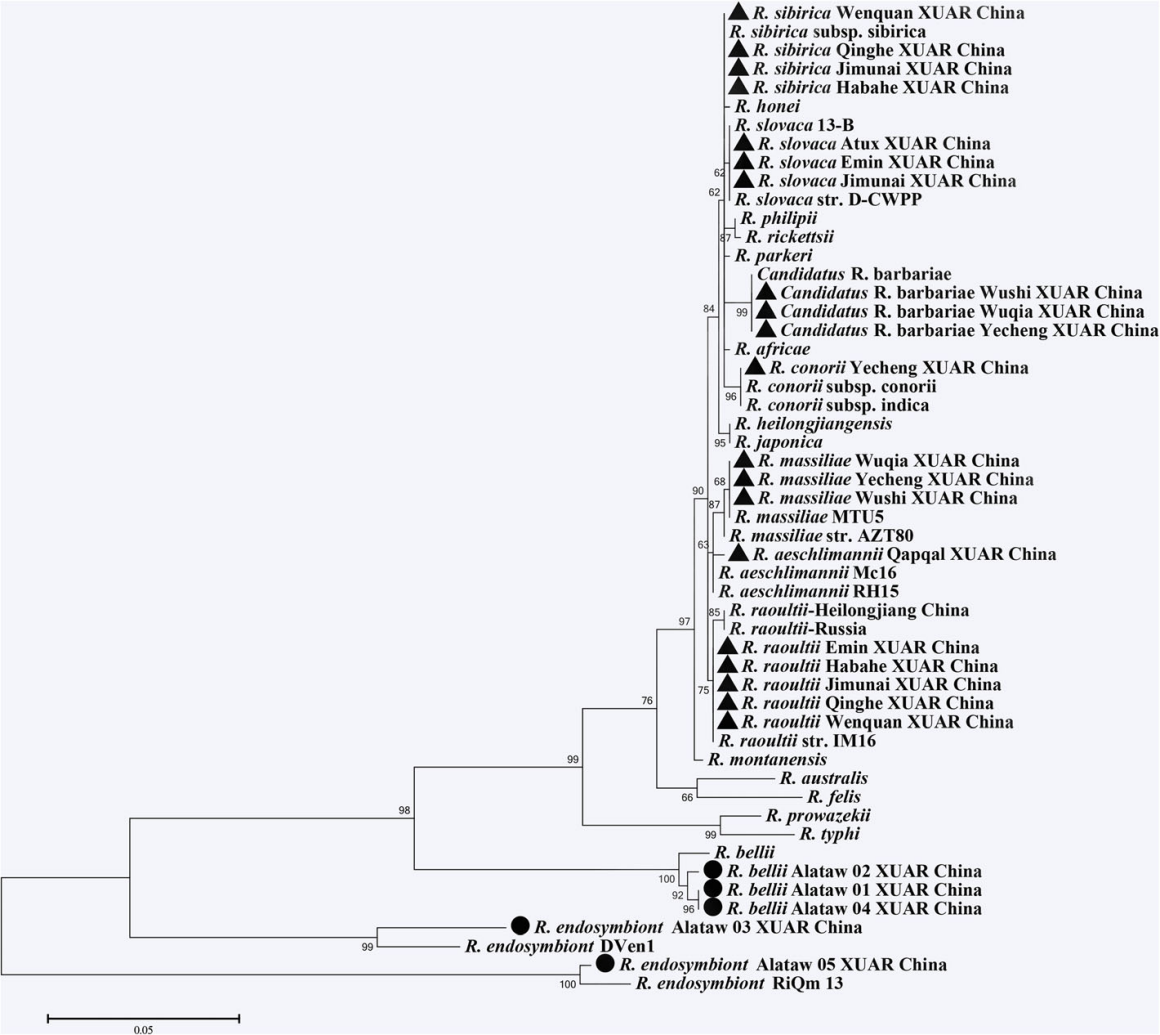
Phylogenic analysis of *Rickettsia* species within ticks and fleas collected from border regions of northwestern China. The tree was constructed with the maximum likelihood (ML; bootstrap replicates: 1000) based on concatenated sequence data for *gltA*-*rrs-17KDa-sca1-ompA-ompB-geneD* genes with MEGA6.0. Sequences of the *Rickettsia* species from ticks and fleas obtained in this study are indicated by triangles and circles, respectively

A total of 982 ticks, belonging to three genera and four species (401 *Rhipicephalus turanicus*, 180 *Dermacentor marginatus*, 319 *D. nuttalli* and 82 *Haemaphysalis punctata*) were collected from ten counties of XUAR. *Rhipicephalus turanicus* (40.84%) was the most frequently collected species, followed by *D. nuttalli* (32.48%), *D. marginatus* (18.33%) and *H. punctata* (8.35%). Seven *Rickettsia* species including *R*. *aeschlimannii*, *R*. *conorii*, *R*. *raoultii*, *Rickettsia sibirica*, *R*. *slovaca*, *R*. *massiliae* and “*Candidatus* R. barbariae” were detected (Table 1, Fig. 2) in the ticks from cattle or sheep. “*Candidatus* R. barbariae” was mainly detected in the southern region, whereas *R. raoultii* was a dominant rickettsial agent in the northern region of XUAR.

**Table 1:** Tick species and PCR results of rickettsiae from questing adult ticks in border regions, northwestern China

Location	Coordinates	Tick species	*N*	*Rickettsia* spp.	No. positive (%)
Qinghe	90°37'E, 46°71'N	*Dermacentor nuttalli*	86	*R. raoultii*	22 (25.58)
				*R. sibirica*	9 (10.46)
Habahe	86°41'E, 48°05'N	*Dermacentor marginatus*	17	*R. raoultii*	3 (17.61)
				*R. sibirica*	1 (5.88)
Jimunai	85°84'E, 47°42'N	*Dermacentor nuttalli*	67	*R. slovaca*	1 (1.49)
				*R. raoultii*	20 (29.85)
				*R. sibirica*	8 (11.94)
Emin	83°62'E, 46°52'N	*Dermacentor marginatus*	60	*R. slovaca*	3 (5.00)
				*R. raoultii*	33 (55.00)
Wenquan	81°08'E, 44°95'N	*Dermacentor nuttalli*	166	*R. raoultii*	57 (34.33)
				*R. sibirica*	8 (4.81)
Qapqal	80°58'E, 43°53'N	*Haemaphysalis punctata*	82	*R. aeschlimannii*	26 (31.70)
Wushi	79°25'E, 41°22'N	*Rhipicephalus turanicus*	180	*R. massiliae*	43 (23.89)
				“*Candidatus* R. barbariae”	47 (26.11)
Atux	76°12'E, 39°73'N	*Dermacentor marginatus*	103	*R. slovaca*	1 (0.97)
				*R. raoultii*	27 (26.21)
Wuqia	75°18'E, 39°07'N	*Rhipicephalus turanicus*	144	*R. massiliae*	46 (31.94)
				“*Candidatus* R. barbariae”	4 (2.78)
Yecheng	77°42'E, 37°89'N	*Rhipicephalus turanicus*	77	*R. conorii*	3 (3.89)
				“*Candidatus* R. barbariae”	34 (44.15)
				*R. massiliae*	23 (29.87)
Total (982)		*Rhipicephalus turanicus*	401	*R. massiliae*	89 (22.19)
				*R. conorii*	3 (0.75)
				“*Candidatus* R. barbariae”	85 (21.20)
		*Dermacentor marginatus*	180	*R. raoultii*	63 (35.00)
				*R. sibirica*	1 (0.56)
				*R. slovaca*	4 (2.22)
		*Dermacentor nuttalli*	319	*R. raoultii*	63 (19.75)
				*R. slovaca*	1 (0.31)
				*R. sibirica*	25 (7.84)
		*Haemaphysalis punctata*	82	*R. aeschlimannii*	26 (31.71)

*Abbreviation*: *N* number of ticks

In addition, a total of 5052 fleas, belonging to 6 families, 15 genera and 18 species were collected from 14 mammalian species, including *Rhombomys opimus*, *Meriones meridianus*, *Meriones libycus*, *Meriones tamariscinus*, *Marmota baibacina*, *Vormela peregusna*, etc. (Table 2). Among them, *Xenopsylla gerbilli minax* (52.40%) was the dominant species, followed by *Paradoxopsyllus repandus* (9.70%), *Citellophilus tesquorum dzetysuensis* (8.31%), *Oropsylla silantiewi* (7.52%) and *Nosopsyllus laeviceps laeviceps* (6.00%). *Rickettsia bellii* and two genotypes of a rickettsia endosymbiont (phylogenetically in an ancestral position to *R. bellii*) were molecularly detected in five flea species (*Echidnophaga oschanin*, *Nosopsyllus laeviceps laeviceps*, *Paradoxopsyllus repandus*, *Rhadinopsylla cedestis* and *Xenopsylla gerbilli minax*) of Alataw county. The BLAST analysis of rickettsial agents are shown in Additional file 2: Table S2.

**Table 2:** The flea species and hosts of origin in this study

Locaton	Coordinates	Flea species	*N*	Flea pool	Host species (*n*)
Alataw	82°33'E, 45°11'N	*Echidnophaga oschanin*	157	15	*Rhombomys opimus (21); *Meriones libycus* (8); *Vormela peregusna* (7)
		*Xenopsylla gerbilli minax*	2647	220	*Rhombomys opimus (44); *Meriones meridianus* (17); *Meriones libycus* (12); *Meriones tamariscinus* (14)
		*Rhadinopsylla cedestis*	20	4	*Meriones tamariscinus (11); *Rhombomys opimus* (3)
		*Paradoxopsyllu*s *repandus*	490	82	*Rhombomys opimus (23); *Meriones meridianus* (8); *Meriones libycus* (11); *Meriones tamariscinus* (7)
		*Nosopsyllus laeviceps laeviceps*	297	45	*Rhombomys opimus (32); *Meriones meridianus* (13); *Meriones libycus* (14); *Meriones tamariscinus* (15)
		*Pulex irritans*	41	8	*Vormela peregusna* (19)
		*Xenopsylla conformis conformis*	118	12	*Rhombomys opimus* (9); *Meriones meridianus (19); *Meriones libycus* (10)
		*Xenopsylla cheopis*	40	8	*Rattus norvegicus* (17)
		*Ctenocephalides felis felis*	140	28	*Felis catus* (32)
		*Coptopsylla lamellifer ardua*	99	35	*Rhombomys opimus (17); *Meriones libycus* (5)
		*Ctenophthalmus dolichus dolichus*	8	1	*Meriones libycus* (3)
		*Pectinoctenus nemorosa*	2	1	*Apodemus sylvaticus* (1); *Mus musculus* (1)
		*Mesopsylla eucta shikno*	4	1	*Allactaga sibirica* (2)
Buerjin	86°92'E, 47°07'N	*Pectinoctenus nemorosa*	8	1	*Apodemus peninsulae* (4)
		*Nosopsyllus laeviceps laeviceps*	6	1	*Rhombomys opimus* (3)
Wenquan	81°08'E, 44°95'N	*Neopsylla mana*	57	7	*Urocitellus undulatus* (27)
		*Frontopsylla elatoides elatoides*	100	16	*Urocitellus undulatus* (34)
		*Oropsylla silantiewi*	380	40	*Marmota baibacina* (55)
		*Citellophilus tesquorum dzetysuensis*	420	50	*Urocitellus undulatus* (53)
Huocheng	80°87'E, 44°07'N	*Xenopsylla gerbilli minax*	10	1	*Rhombomys opimus* (1)
Qapqal	80°58'E, 43°53'N	*Ischnopsyllus octactenus*	8	1	*Pipistrellus pipistrellus* (4)
Total 5052 (577)		*Xenopsylla gerbilli minax*	2657	221	

*Abbreviations*: *N* number of fleaa, *n* number of hosts, * main host

## Discussion

In this study, nine *Rickettsia* species have been molecularly detected in 982 ticks and 5052 fleas collected in 13 border counties of XUAR, neighboring Kyrgyzstan, Mongolia, Pakistan, Russia and Kazakhstan. Considering ticks, *R. massiliae*, *R. aeschlimannii* and “*Candidatus* R. barbariae” have been detected in *Rh. turanicus*, while *R. raoultii*, *R. slovaca* and *R. sibirica* were found in *D. marginatus* and *D. nuttalli*. Based on these findings, several SFG *Rickettsia* species occur in highly abundant tick species in the border regions of XUAR. In particular, *Rh. turanicus*, *D. marginatus* and *D. nuttalli* might play key roles in the propagation of rickettsiae across country borders in the region. In addition, the present data revealed significant differences in the spectrum and prevalence of rickettsiae between the north and south XUAR, most likely as a consequence of variations in the abundance of corresponding vectors and reservoirs.

*Rickettsia bellii* was previously detected in members of the genera *Dermacentor* and *Amblyomma*, in which it also undergoes transovarial transmission [30]. This *Rickettsia* species can be cultured in mammalian cells and may cause disease in mammals [30]. Here, *R. bellii* is reported for the first time in China. Detections of *R. bellii* in *Xenopsylla gerbilli minax*, *Echidnophaga oschanin* and *Paradoxopsyllus repandus* fleas are also novel findings. More interestingly, two genotypes of a rickettsia endosymbiont (ancestral to *R. bellii*) have been identified for the first time in *Rhadinopsylla cedestis* and *Nosopsyllus laeviceps laeviceps* fleas. These results indicate that some flea species infesting wild rodents in the border regions might carry different, probably ancient *Rickettsia* species or genotypes. Therefore, these data extend our knowledge on the geographical distribution and reservoir spectrum of AG rickettsiae.

XUAR has a great variety of landscape and habitats, maintaining a broad range of mammalian and avian species, which could serve as hosts of diverse tick and flea species. In this study, seven SFG and two AG *Rickettsia* species have been detected in border regions of northwest China. Surveillances of rickettsial agents in border regions of Central Asia are particularly useful and informative, because data from such monitoring studies are relevant to several countries, which are involved in international trade of livestock and livestock products, but may also be affected (in the context of rickettsioses) by movements of wildlife and migratory birds [11, 31].

## Conclusions

In this study, nine *Rickettsia* species were molecularly detected in 982 ticks and 5052 fleas in the border regions of XUAR, northwestern China. The data indicate the occurrence of several SFG rickettsiae in *Rh. turanicus*, *D. nuttalli* and *D. marginatus* collected from ruminants, as well as of AG rickettsiae in fleas infesting wild rodents. These data extend our knowledge on the diversity of and potential vector/reservoir range of rickettsiae in central Asia.

## Additional files

Additional file 1: **Table S1.** GenBank accession numbers of representative nucleotide sequences, including 22 from ticks, 78 from fleas, 84 from rickettsiae in ticks and 12 from rickettsiae in fleas, are shown in A, B, C and D, respectively. (DOCX 45 kb)

Additional file 2: **Table S2.** *Rickettsia* endosymbiont and *Rickettsia bellii* detected in this study. (DOCX 15 kb)

## Abbreviations

17-*kDa*: 17-kDa antigen
*cox*1: cytochrome *c* oxidase subunit 1
*gene* D: PS120-proteinencoding gene
*glt*A: citrate synthase
*omp*A: outer membrane proteins A
*omp*B: outer membrane proteins B
*rrs*: *16S* rRNA gene
*sca*1: cell surface antigen 1
SFG: spotted fever group
XUAR: Xinjiang Uygur Autonomous Region
