# Supplementary material for: Diversity of Rickettsia species in border regions of northwestern China
Source: Parasit Vectors. 2018 Dec 13;11:634. doi: 10.1186/s13071-018-3233-6 (PMC6293579; doi:10.1186/s13071-018-3233-6)
Supplement: Supplementary file 1 — Table S1. GenBank accession numbers of representative nucleotide sequences, including 22 from ticks, 78 from fleas, 84 from rickettsiae in ticks and 12 from rickettsiae in fleas, are shown in A, B, C and D, respectively. (DOCX 45 kb) [file 13071_2018_3233_MOESM1_ESM.docx]

**Additional file 1: Table S1.** GenBank accession numbers of representative nucleotide sequences, including 22 from ticks, 78 from fleas, 84 from rickettsiae in ticks and 12 from rickettsiae in fleas, are shown in sections A, B, C and D, respectively.

| **Location** | **Gene** | **Accession number** | | **Reference sequence** | **Similarity % (bp)** |
| --- | --- | --- | --- | --- | --- |
| **A** | | | | | |
| ***Rhipicephalus turanicus*** | | | | | |
| Yecheng | *16S rRNA* | KU364375 | | *Rh. turanicus* FK-1 (KY583075) | 99.26 (405/408) |
|  | *cox*1 | MF002578 | | *Rh. turanicus* FK-1 (KY583075) | 100 (889/889) |
| Wushi | *16S rRNA* | MF002560 | | *Rh. turanicus* YC1 (KY583077) | 100 (453/453) |
|  | *12S rRNA* | MF002567 | | *Rh. turanicus* Shaya4 (KR809580) | 100 (400/400) |
|  | *cox*1 | MF002580 | | *Rh. turanicus* Alsk-1 (KY606288) | 99.77 (887/889) |
| Wuqia | *16S rRNA* | MF002558 | | *Rh. turanicus* TMSK6-1 (KY583076) | 100 (455/455) |
|  | *12S rRNA* | MF002568 | | *Rh. turanicus* Akesu (KR809575) | 100 (400/400) |
|  | *cox*1 | MF002581 | | *Rh. turanicus* Alsk-1 (KY606288) | 99.77 (887/889) |
| ***Haemaphysalis punctata*** | | | | | |
| Qapqal | *16S rRNA* | MF002565 | *Ha. punctata* Orkun-HAPU21（KR870978) | | 100 (455/455) |
|  | *12S rRNA* | MF002576 | *Ha. punctata* (AM410575) | | 100 (338/338) |
| ***Dermacentor marginatus*** | | | | | |
| Atux | *16S rRNA* | MF002563 | *D. marginatus* WQ-DM54 (KX555656) | | 100 (433/433) |
|  | *12S rRNA* | MF002570 | *D. marginatus* (AM410570) | | 100 (382/382) |
| Emin | *16S rRNA* | MF002562 | *D. marginatus* WQ-DM54 (KX555656) | | 99.07 (429/433) |
|  | *12S rRNA* | MF002572 | *D. marginatus* (AM410570) | | 100 (382/382) |
| Habaha | *16S rRNA* | MF002564 | *D. marginatus* WQ-DM54 (KX555656) | | 99.07 (429/433) |
|  | *12S rRNA* | MF002571 | *D. marginatus* (AM410570) | | 100 (382/382) |
| ***Dermacentor nuttalli*** | | | | | |
| Qinghe | *16S rRNA* | MF002561 | *D. nuttalli* XJ088 (JX05111) | | 100 (456/456) |
|  | *12S rRNA* | MF002573 | *D. nuttalli* (KT764942) | | 100 (382/382) |
| Wenquan | *16S rRNA* | MF002561 | *D. nuttalli* XJ088 (JX051114) | | 100 (456/456) |
|  | *12S rRNA* | MF002574 | *D. nuttalli* (KT764942) | | 100 (382/382) |
| Jimunai | *16S rRNA* | MF002561 | *D. nuttalli* XJ088 (JX051114) | | 99.34 (453/456) |
|  | *12S rRNA* | MF002575 | *D. nuttalli* (KT764942) | | 100 (382/382) |
| **B** | | | | | |
| Alataw | *18S rDNA* | KX668367 | *Echidnophaga oschanini* (KM891225) | | 100 (380/380) |
|  |  | KX999716 | *P. irritans* (AF423915) | | 100 (1152/1152) |
|  |  | KX668365 | *X. conformis conformis* (KM891229) | | 100 (410/410) |
|  |  | KX668366 | *X. cunicularis (EU336098)* | | 99.3 (1145/1153) |
|  |  | KX999715 | *C. felis (KC177274)* | | 100 (1152/1152) |
|  |  | KX668370 | *Co. africana (AF286275)* | | 99.3 (1144/1152) |
|  |  | KX668369 | *Ct. formosanus (EU336131)* | | 100 (1151/1151) |
|  |  | KX668368 | *Rh. difficilis (EU336044)* | | 99.2 (1143/1152) |
|  |  | KX668372 | *P. nemorosa (KM891153)* | | 98.9 (1126/1138) |
|  |  | KX668373 | *M. hebes clara (KM891216)* | | 100 (383/383) |
|  |  | KX668371 | *Geusibia ashcrafti (EU336095)* | | 99.7 (1148/1152) |
|  |  | KX668374 | *No. iranus theodori (EU336051)* | | 99.7 (1149/1152) |
| Burqin |  | KY593307 | *P. nemorosa (KM891153)* | | 98.9 (1126/1138) |
|  |  | KY593308 | *No. laeviceps ellobii (KM891222)* | | 96.6 (370/383) |
| Wenquan |  | KY593301 | *Ne. mana (KM891218)* | | 98.6 (729/739) |
|  |  | KY593303 | *F. spadix (DQ298443)* | | 99.9 (1150/1151) |
|  |  | KT878385 | *O. montana (EU336048)* | | 99.8 (1150/1152) |
|  |  | KY593298 | *C. tesquorum mongolicus* (EU336103) | | 99.8 (1150/1152) |
| Huocheng |  | KY593306 | *X. ramesis (KM891146)* | | 98.3 (1133/1153) |
| Qapqal |  | KY593300 | *I. variabilis (KM891200)* | | 98.7 (1136/1105) |
| Alataw | *28s rDNA* | KX668377 | *E. iberica (EU336207)* | | 99.5 (995/1000) |
|  |  | KX999718 | *P. irritans (AF423975)* | | 100 (998/998) |
|  |  | KX668375 | *X. cunicularis (EU336206)* | | 98.4 (986/1002) |
|  |  | KX668376 | *X. cunicularis (EU336206)* | | 98.6 (988/1002) |
|  |  | KY593316 | *X. cheopis (EU336145)* | | 100 (1001/1001) |
|  |  | KX999717 | *C. felis (EU414732)* | | 100 (997/997) |
|  |  | KX668380 | *Co. africana (AF423945)* | | 98.7 (992/1005) |
|  |  | KX668379 | *Ct. formosanus (EU336238)* | | 99.5 (986/991) |
|  |  | KX668378 | *Rh. difficilis (EU336151)* | | 98.8 (984/996) |
|  |  | KX668382 | *Ct. formosanus (EU336238)* | | 94.2 (949/1007) |
|  |  | KX668383 | *M. hebes clara (KM891099)* | | 88.2 (893/993) |
|  |  | KX668381 | *F. nakagawai borealosinica (EU336181)* | | 99.4 (987/993) |
|  |  | KX668384 | *No. iranus theodori (EU336159)* | | 99.3 (984/991) |
| Burqin |  | KY593319 | *No. laeviceps ellobii (KM891105)* | | 90.5 (847/936) |
|  |  | KY593307 | *P. nemorosa (KM891153)* | | 98.9 (1126/1138) |
| Wenquan |  | KY593314 | *F. nakagawai borealosinica (EU336181)* | | 99.5 (988/993) |
|  |  | KT878386 | *O. montana (EU336156)* | | 98.5 (978/993) |
|  |  | KY593309 | *C. tesquorum mongolicus (EU336211)* | | 99.9 (992/993) |
|  |  | KY593312 | *Ne. bidentatiformis (EU336182)* | | 99.6 (988/992) |
| Huocheng |  | KY593317 | *X. cheopis (EU336145)* | | 92.7 (930/1003) |
| Qapqal |  | KY593311 | *Myodopsylla palposa (EU336162)* | | 99.1 (983/992) |
| Alataw | *cox*2 | MF045759 | *E. oschanini (KU880675)* | | 100 (702/702) |
|  |  | MF136072 | *Pulex sp.（KM890886）* | | 96.4 (619/642) |
|  |  | MF136073 | *X. conformis conformis （KM890859）* | | 100 (678/678) |
|  |  | MF136074 | *X. gerbilli minax (KU880677)* | | 100 (683/683) |
|  |  | MF000679 | *X. cheopis (KJ638529)* | | 100 (609/609) |
|  |  | MF136071 | *C. felis (KF684933)* | | 99.6 (706/709) |
|  |  | MF045762 | *Co. africana (AF424009)* | | 89.2 (544/610) |
|  |  | MF045761 | *Ct. formosanus (EU336024)* | | 91.0 (555/610) |
|  |  | MF045760 | *Rh. dahurica dahurica (KM890784)* | | 89.0 (581/653) |
|  |  | MF045764 | *P. pavlovskii (KM890857)* | | 90.7 (596/657) |
|  |  | MF045765 | *M. lenis (KM890797)* | | 97.6 (639/655) |
|  |  | MF045763 | *F. hetera (KM890840)* | | 91.1 (613/673) |
|  |  | MF045767 | *No. laeviceps ellobii (KM890856)* | | 100 (675/675) |
| Burqin |  | MF000680 | *P. nemorosa (KM890779)* | | 99.7 (649/651) |
|  |  | MF000681 | *No. laeviceps ellobii (KM890856)* | | 99.9 (677/678) |
| Wenquan |  | MF000671 | *F. hetera (KM890840)* | | 97.5 (661/678) |
|  |  | KU601752 | *O. hirsuta (KM890877)* | | 95.8 (635/663) |
|  |  | MF000668 | *C. tesquorum mongolicus (KM890842)* | | 94.1 (610/648) |
|  |  | MF000670 | *Ne. mana (AF257461)* | | 98.4 (716/728) |
| Huocheng |  | MF136074 | *X. gerbilli minax (KU880677)* | | 100 (683/683) |
| Qapqal |  | MF000678 | *I. indicus (KM890860)* | | 90.6 (590/651) |
| Alataw | *EF1-a* | KX822751 | *E. iberica (EU336310)* | | 97.3 (805/827) |
|  |  | KX822749 | *X. conformis conformis (KM890553)* | | 100 (812/812) |
|  |  | KX822750 | *X. skrjabini (KM890548)* | | 96.6 (796/824) |
|  |  | KX999719 | *C. canis (AF423870)* | | 99.4 (822/827) |
|  |  | KX822754 | *Co. africana (AF423838)* | | 93.8 (776/827) |
|  |  | KX822752 | *Rh. heiseri (EU336246)* | | 86.2 (713/827) |
|  |  | KX822755 | *P. nemorosa (KM890482)* | | 99.8 (813/815) |
|  |  | KX822756 | *M. hebes clara (KM890541)* | | 97.1 (800/824) |
|  |  | KX822757 | *No. laeviceps laeviceps (EU336292)* | | 99.3 (821/827) |
| Burqin |  | KY610544 | *P. lauta (KM890481)* | | 96.9 (790/815) |
|  |  | KY610545 | *No. iranus theodori (EU336264)* | | 98.5 (809/821) |
| Wenquan |  | KY610535 | *Ne. bidentatiformis (EU336287)* | | 97.0 (799/824) |
|  |  | KY610537 | *F. nakagawai borealosinica (EU336286)* | | 94.4 (781/827) |
|  |  | KT878387 | *O. agilis (EU336243)* | | 94.8 (784/827) |
|  |  | KY610534 | *C. sparsilis (AF423834)* | | 97.1 (803/827) |
| Huocheng |  | KY610543 | *X. conformis conformis (KM890553)* | | 94.8 (781/824) |
| Qapqal |  | KY610542 | *I. variabilis (KM890525)* | | 96.8 (798/824) |
| **C** | | | | | |
| ***Rickettsia raoultii*** | | | | | |
| Qinghe, Jimunai, Wenquan | *rrs* | MF002585 | *R. raoultii* str. Khabarovsk (CP010969) | | 100 (1284/1284) |
|  | *17kDa* | MF002524 | *R. raoultii* str. Khabarovsk (CP010969) | | 100 (434/434) |
|  | *gltA* | MF002515 | *R. raoultii* str. Khabarovsk (CP010969) | | 100 (1178/1178) |
|  | *ompA* | MF002522 | *R. raoultii* str. Khabarovsk (CP010969) | | 100 (528/528) |
|  | *ompB* | MF002526 | *R. raoultii* str. Khabarovsk (CP010969) | | 100 (812/812) |
|  | *sca1* | MF002520 | *R. raoultii* str. Khabarovsk (CP010969) | | 100 (657/657) |
|  | *geneD* | MF002518 | *R. raoultii* str. Khabarovsk (CP010969) | | 100 (902/902) |
| Habaha, Emin | *rrs* | MF002586 | *R. raoultii* str. Khabarovsk (CP010969) | | 100 (1284/1284) |
|  | *17kDa* | MF002525 | *R. raoultii* str. Khabarovsk (CP010969) | | 100 (434/434) |
|  | *gltA* | MF002516 | *R. raoultii* str. Khabarovsk (CP010969) | | 100 (1178/1178) |
|  | *ompA* | MF002523 | *R. raoultii* str. Khabarovsk (CP010969) | | 100 (528/528) |
|  | *ompB* | MF002527 | *R. raoultii* str. Khabarovsk (CP010969) | | 100 (812/812) |
|  | *sca1* | MF002521 | *R. raoultii* str. Khabarovsk (CP010969) | | 100 (657/657) |
|  | *geneD* | MF002519 | *R. raoultii* str. Khabarovsk (CP010969) | | 100 (902/902) |
| ***Rickettsia massiliae*** | | | | | |
| Yecheng | *rrs* | KU364357 | *R. massiliae* str. MTU5 (CP000683) | | 100 (1182/1182) |
|  | *17kDa* | KU364359 | *R. massiliae* str*.* MTU5 (CP000683) | | 99.74 (391/392) |
|  | *gltA* | KU364354 | *R. massiliae* str. MTU5 (CP000683) | | 100 (1118/1118) |
|  | *ompA* | KU757305 | *R. massiliae* str. MTU5 (CP000683) | | 100 (491/491) |
|  | *ompB* | KU364370 | *R. massiliae* str. MTU5 (CP000683) | | 99.86 (765/766) |
|  | *sca1* | KU757304 | *R. massiliae* str*.* MTU5 (CP000683) | | 100 (611/611) |
|  | *geneD* | KU757306 | *R. massiliae* str. MTU5 (CP000683) | | 100 (900/900) |
| Wushi, Wuqia | *rrs* | MF002582 | *R. massiliae* str. MTU5 (CP000683) | | 100 (1284/1284) |
|  | *17kDa* | MF002501 | *R. massiliae* str*.* MTU5 (CP000683) | | 100 (434/434) |
|  | *gltA* | MF002497 | *R. massiliae* str*.* MTU5 (CP000683) | | 100 (1178/1178) |
|  | *ompA* | MF002500 | *R. massiliae* str*.* MTU5 (CP000683) | | 100 (532/532) |
|  | *ompB* | MF002502 | *R. massiliae* str*.* MTU5 (CP000683) | | 100 (812/812) |
|  | *sca1* | MF002499 | *R. massiliae* str*.* MTU5 (CP000683) | | 100 (657/657) |
|  | *geneD* | MF002498 | *R. massiliae* str. MTU5 (CP000683) | | 100 (920/920) |
| ***Rickettsia sibirica*** | | | | | |
| Habaha | *rrs* | MF002589 | *R. sibirica* RH15 (HM050271) | | 100 (1284/1284) |
|  | *17kDa* | MF002548 | *R. sibirica* (AF445384) | | 100 (434/434) |
|  | *gltA* | MF002540 | *R. sibirica* subsp. sibirica (KM288711) | | 100 (1178/1178) |
|  | *ompA* | MF002546 | *R. sibirica* subsp. sibirica (KM288712) | | 99.81 (531/532) |
|  | *ompB* | MF002550 | *R. sibirica* BJ-90 (AY331393) | | 100 (812/812) |
|  | *sca1* | MF002544 | *R. sibirica* ATCC VR-151T (AY355356) | | 100 (657/657) |
|  | *geneD* | MF002542 | *R. sibirica* (AF155057) | | 100 (910/910) |
| Qinghe, Jimunai, Wenquan | *rrs* | MF002590 | *R. sibirica* RH15 (HM050271) | | 100 (1284/1284) |
|  | *17kDa* | MF002549 | *R. sibirica* (AF445384) | | 100 (434/434) |
|  | *gltA* | MF002541 | *R. sibirica* subsp. sibirica (KM288711) | | 100 (1178/1178) |
|  | *ompA* | MF002547 | *R. sibirica* subsp. sibirica (KM288712) | | 99.81 (531/532) |
|  | *ompB* | MF002551 | *R. sibirica* BJ-90 (AY331393) | | 100 (812/812) |
|  | *sca1* | MF002545 | *R. sibirica* ATCC VR-151T (AY355356) | | 100 (657/657) |
|  | *geneD* | MF002543 | *R. sibirica* (AF155057) | | 100 (910/910) |
| ***Rickettsia aeschlimannii*** | | | | | |
| Qapqal | *rrs* | MF002591 | *R. aeschlimannii* RH15 (HM050274) | | 99.74 (1196/1199) |
|  | *17kDa* | MF002556 | *R. aeschlimannii* (CP019435) | | 99.28 (419/422) |
|  | *gltA* | MF002552 | *R. aeschlimannii* (AY259084) | | 99.91 (1116/1117) |
|  | *ompA* | MF002555 | *R. aeschlimannii* (JN944634) | | 98.58 (486/493) |
|  | *ompB* | MF002557 | *R. rhipicephali* (CP013133) | | 99.11 (781/788) |
|  | *sca1* | MF002554 | *R. aeschlimannii* (AY355353) | | 99.35 (618/622) |
|  | *geneD* | MF002553 | *R. aeschlimannii* RH15 (HM050274) | | 98.19 (868/884) |
| ***Rickettsia conorii*** | | | | | |
| Yecheng | *rrs* | KU364355 | *R. conorii* subsp. conorii (U43794) | | 100 (1185/1185) |
|  | *17kDa* | KU364361 | *R. conorii* (M28480) | | 100 (410/410) |
|  | *gltA* | KU364366 | *R. conorii* Malish 7 (AE006914) | | 100 (1076/107) |
|  | *ompA* | KU364365 | *R. conorii* subsp. conorii (U43794) | | 100 (451/451) |
|  | *ompB* | KU364371 | *R. conorii* subsp. conorii (AF123726) | | 99.60 (763/766) |
|  | *ompB^1^* | KU757301 | *R. conorii* subsp. conorii (AF123726) | | 100 (1000/1000) |
|  | *sca1* | KU364363 | *R. conorii* Malish 7 (AE006914) | | 99.84 (625/626) |
|  | *geneD* | KU757303 | *R. conorii* ATCC VR-597 (AF163005) | | 100 (897/897) |
| **Candidatus Rickettsia barbariae** | | | | | |
| Wushi | *rrs* | KU364356 | Candidatus R. barbariae (EU272189) | | 100 (1174/1174) |
|  | *17kDa* | KU364358 | Candidatus R. barbariae (GU353184) | | 100 (399/399) |
|  | *gltA* | KU364367 | *R. sibirica* (KM28871) | | 99.54 (1096/1101) |
|  | *ompA* | KU364364 | Candidatus R. barbariae (EU272186) | | 100 (436/436) |
|  | *ompB* | KU364369 | 1. *parkeri* str. Portsmouth (CP003341) | | 99.47 (751/755) |
|  | *sca1* | KU364362 | *R. africae* str. ESF-5 (CP001612) | | 99.52 (622/625) |
|  | *geneD* | KU757302 | Candidatus R. barbariae ( EU272188) | | 100 (917/917) |
| Wuqia, Yecheng | *rrs* | MF002583 | Candidatus R. barbariae (EU272189) | | 100 (1284/1284) |
|  | *17kDa* | MF002507 | Candidatus R. barbariae (GU353184) | | 100 (399/399) |
|  | *gltA* | MF002503 | *R. sibirica* (KM28871) | | 99.54 (1096/1101) |
|  | *ompA* | MF002506 | Candidatus R. barbariae (EU194445) | | 100 (509/509) |
|  | *ompB* | MF002508 | 1. *parkeri* str. Portsmouth (CP003341) | | 97.46 (806/827) |
|  | *sca1* | MF002505 | *R. africae* str. ESF-5 (CP001612) | | 99.39 (654/658) |
|  | *geneD* | MF002504 | Candidatus R. barbariae (KU645286) | | 100 (920/920) |
| ***Rickettsia slovaca*** | | | | | |
| Atux, Jimunai | *rrs* | MF002587 | *R. slovaca* str. D-CWPP (CP003375) | | 100 (1284/1284) |
|  | *17kDa* | MF002536 | *R. slovaca* str. D-CWPP (CP003375) | | 100 (434/434) |
|  | *gltA* | MF002528 | *R. slovaca* str. D-CWPP (CP003375) | | 100 (1178/1178) |
|  | *ompA* | MF002534 | *R. slovaca* str. D-CWPP (CP003375) | | 100 (533/533) |
|  | *ompB* | MF002538 | *R. slovaca* str. D-CWPP (CP003375) | | 100 (812/812) |
|  | *sca1* | MF002532 | *R. slovaca* str. D-CWPP (CP003375) | | 100 (657/657) |
|  | *geneD* | MF002530 | *R. slovaca* str. D-CWPP (CP003375) | | 100 (920/920) |
| Emin | *rrs* | MF002588 | *R. slovaca* str. D-CWPP (CP003375) | | 100 (1284/1284) |
|  | *17kDa* | MF002537 | *R. slovaca* str. D-CWPP (CP003375) | | 100 (434/434) |
|  | *gltA* | MF002529 | *R. slovaca* str. D-CWPP (CP003375) | | 100 (1178/1178) |
|  | *ompA* | MF002535 | *R. slovaca* str. D-CWPP (CP003375) | | 100 (533/533) |
|  | *ompB* | MF002539 | *R. slovaca* str. D-CWPP (CP003375) | | 100 (812/812) |
|  | *sca1* | MF002533 | *R. slovaca* str. D-CWPP (CP003375) | | 100 (657/657) |
|  | *geneD* | MF002531 | *R. slovaca* str. D-CWPP (CP003375) | | 100 (920/920) |
| **D** | | | | | |
| ***Rickettsia endosymbiont*** | | | | | |
| Alataw | *rrs* | KX457947 | *Rickettsia* sp. (AB021128) | | 99.46 (1278/1285) |
|  |  | KX457949 | *R. bellii* str. RML369-C (NR074484) | | 99.31 (1154/1162) |
|  | *gltA* | KX457951 | *R. bellii* RML369-C (CP000087) | | 98.90 (811/820) |
|  |  | KX457954 | *R. endosymbiont* str. 250 (JQ925616) | | 99.26 (405/408) |
| ***Rickettsia bellii*** | | | | | |
| Alataw | *rrs* | KX457946 | *R. bellii* str. RML369-C (NR074484) | | 99.83 (1166/1168) |
|  |  | KX457948 | *R. bellii* str. RML369-C (NR074484) | | 99.83 (1166/1168) |
|  |  | KX254161 | *R. bellii* str. RML369-C (NR074484) | | 99.84 (1282/1284) |
|  | *gltA* | KX457950 | *R. bellii* RML369-C (CP000087) | | 98.97 (771/779) |
|  |  | KX457952 | *R. endosymbiont* G citrate (FJ666753) | | 95.06 (673/708) |
|  |  | KX457953 | *R. bellii* RML369-C (CP000087) | | 98.78 (810/820) |
|  | *17KDa* | KX254163 | *R. bellii* OSU 85-389 (CP000849) | | 99.42 (515/518) |
|  |  | KX254164 | *R. bellii* OSU 85-389 (CP000849) | | 99.23 (514/518) |
