# Supplementary material for: Diversity of Rickettsia species in border regions of northwestern China
Source: Parasit Vectors. 2018 Dec 13;11:634. doi: 10.1186/s13071-018-3233-6 (PMC6293579; doi:10.1186/s13071-018-3233-6)
Supplement: Supplementary file 2 — Table S2. Rickettsia endosymbiont and Rickettsia bellii detected in this study. (DOCX 15 kb) [file 13071_2018_3233_MOESM2_ESM.docx]

**Additional file 2: Table S2.** *Rickettsia* endosymbiont and *Rickettsia bellii* detected in this study

| ***Rickettsia endosymbiont* detected in this study** | | | | |
| --- | --- | --- | --- | --- |
| Origin of flea species | Target genes | Accession numbers | Reference sequence | Similarity % (bp) |
| *Rhadinosylla cedestis* | *rrs* | KX457947 | *Rickettsia* sp. (AB021128) | 99.46% (1278/1285) |
| *Nosopsyllus laeviceps laeviceps* |  | KX457949 | *R. bellii* str. RML369-C (NR074484) | 99.31% (1154/1162) |
| *Echidnophaga oschanini* | *gltA* | KX457951 | *R. bellii* RML369-C (CP000087) | 98.90% (811/820) |
| *Nosopsyllus laeviceps laeviceps* |  | KX457954 | *R. endosymbiont* str. 250 (JQ925616) | 99.26% (405/408) |
| ***Rickettsia bellii* detected in this study** | | | | |
| *Echidnophaga oschanini* | *rrs* | KX457946 | *R. bellii* str. RML369-C (NR074484) | 99.83% (1166/1168) |
| *Paradoxopsyllus repandus* |  | KX457948 | *R. bellii* str. RML369-C (NR074484) | 99.83% (1166/1168) |
| *Xenopsylla gerbilli minax* |  | KX254161 | *R. bellii* str. RML369-C (NR074484) | 99.84% (1282/1284) |
| *Xenopsylla gerbilli minax* | *gltA* | KX457950 | *R. bellii* RML369-C (CP000087) | 98.97% (771/779) |
| *Rhadinopsylla cedestis* |  | KX457952 | *R. endosymbiont* G citrate (FJ666753) | 95.06% (673/708) |
| *Paradoxopsyllus repandus* |  | KX457953 | *R. bellii* RML369-C (CP000087) | 98.78% (810/820) |
| *Xenopsylla gerbilli minax* | *17KDa* | KX254163 | *R. bellii* OSU 85-389 (CP000849) | 99.42% (515/518) |
| *Xenopsylla gerbilli minax* |  | KX254164 | *R. bellii* OSU 85-389 (CP000849) | 99.23% (514/518) |
